# Crohn’s Disease Requiring Different Surgical Interventions in Presumed Monozygotic Twin Brothers: A Case Report

**DOI:** 10.70352/scrj.cr.26-0663

**Published:** 2026-11-05

**Authors:** Keigo Matsunaga, Keiji Matsuda, Tatsuki Kotani, Kentaro Abe, Rie Kondo, Kiyohiko Omichi, Takeshi Nishikawa, Wataru Masuda

**Affiliations:** 1 Department of Surgery, The Fraternity Memorial Hospital, Tokyo, Japan; 2 Department of Pathology, The Fraternity Memorial Hospital, Tokyo, Japan

**Keywords:** Crohn’s disease, twins, perianal fistula, intestinal stenosis, surgery

## Abstract

**INTRODUCTION:**

Crohn’s disease (CD) in both members of a presumed monozygotic twin pair is uncommon. We report twin brothers who developed ileocolonic and perianal CD 2 years apart but required different surgical interventions. This report highlights the value of prompt evaluation of a symptomatic cotwin and individualized surgical decision-making.

**CASE PRESENTATION:**

Twin 1 developed a perianal abscess at the age of 16 years, followed by severe rectosigmoid inflammation and fixed stenosis. Endoscopy demonstrated rectosigmoid mucosal necrosis, ileocecal scarring, and longitudinal ulcer scars in the small bowel, and biopsy specimens contained epithelioid granulomas. Recurrent obstructive symptoms led to an anterior resection. Twin 2 developed a perianal abscess at the age of 18 years. Because his cotwin had CD, he underwent early ileocolonoscopy, which showed longitudinal ulcers extending from the terminal ileum to the cecum and aphthous ulcers throughout the colorectum. A symptomatic low intersphincteric fistula was treated by seton drainage, and curetted tissue contained epithelioid granulomas. Both brothers initially received vedolizumab after surgery. All seton-related drains in Twin 2 were removed 3 months postoperatively. Drainage from the fistula recurred at 6 months, prompting a switch from vedolizumab to adalimumab; the drainage ceased 1 month later. Zygosity was reported by the patients and their family but was not genetically confirmed. Twin 1 remained free of recurrent obstruction for more than 2 years.

**CONCLUSIONS:**

This rare presumed monozygotic twin pair showed close concordance in the age at onset, ileocolonic and perianal involvement, and granulomatous histology, while requiring different operations and postoperative medical strategies. Their clinical courses underscore the importance of asking about family history, promptly investigating gastrointestinal or perianal symptoms in a cotwin, and individualizing treatment over time. Further accumulation of well-characterized twin cases with objective zygosity assessment and long-term follow-up may clarify the shared and divergent clinical features of CD.

## Abbreviation

CD
: Crohn’s disease

## INTRODUCTION

CD is a chronic inflammatory disorder that arises from interactions among genetic susceptibility, environmental exposures, the intestinal microbiota, and dysregulated mucosal immunity.^1,2)^ The higher concordance of CD in monozygotic twins than in dizygotic twins supports a genetic contribution, whereas incomplete concordance indicates that genetic background alone does not determine disease development or phenotype.^3,4)^ Reports of concordant CD in presumed monozygotic twins remain uncommon in East Asia. We report twin brothers who developed ileocolonic and perianal CD 2 years apart and required different surgical interventions. Rather than attributing differences in their early clinical courses to specific genetic or environmental factors, we focus on the practical surgical lessons: prompt investigation of a symptomatic cotwin and phenotype-based selection of treatment for each patient.

## CASE PRESENTATION

The pedigree is shown in **Fig. 1**. The patients and their family reported that the brothers were monozygotic twins; however, zygosity testing was not performed. No other family member shown in the pedigree had been diagnosed with inflammatory bowel disease. Both brothers were nonsmokers and students. They lived in the same family home, had no substantial differences in diet, shared the same childhood living environment, and did not routinely use antibiotics. Before their CD-related presentations, neither brother had a notable history of infection.

**Fig. 1:**
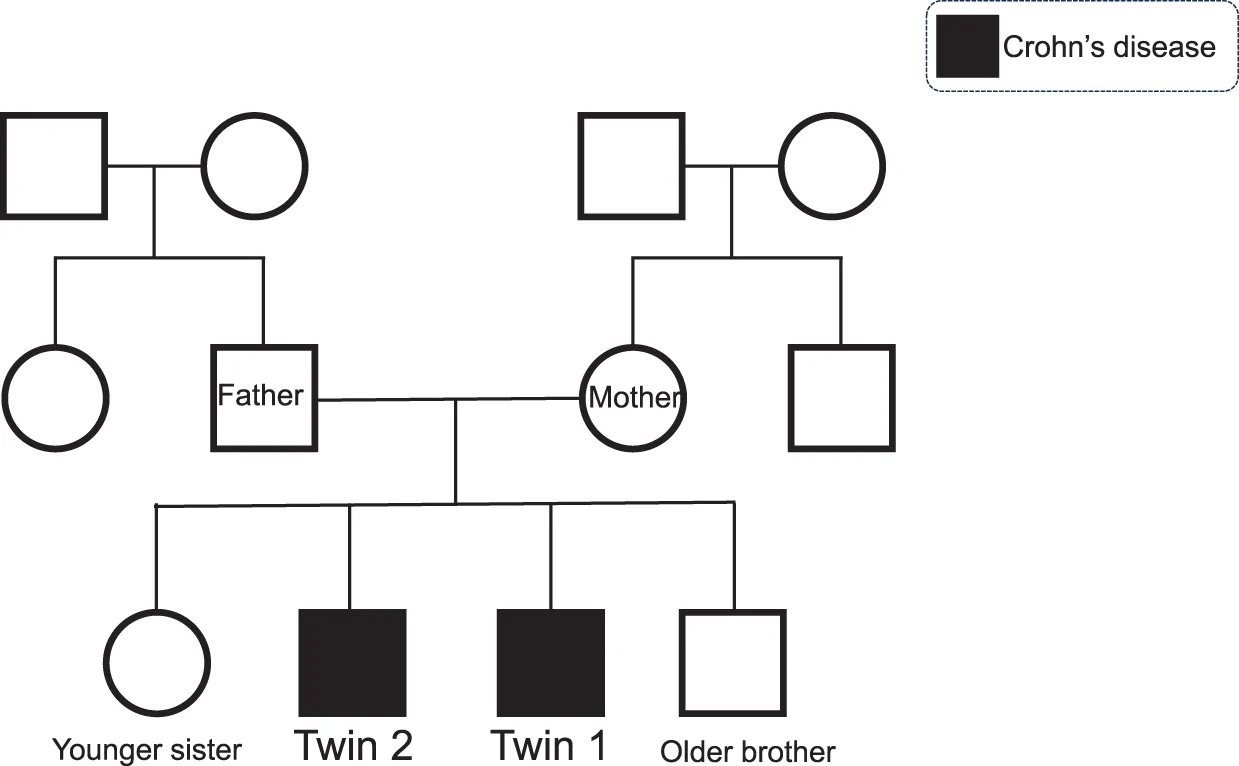
Pedigree of the affected twin brothers. Filled symbols indicate individuals with CD. No other family member shown had been diagnosed with inflammatory bowel disease. CD, Crohn’s disease

### Twin 1

The elder brother had no relevant medical history when, at the age of 16 years, he underwent incision and drainage of a perianal abscess at another hospital in April 20XX. In July, he was admitted elsewhere because of abdominal pain and hematochezia. Colonoscopy showed continuous severe inflammation extending from the rectum to the descending colon, prompting suspicion of CD. Corticosteroid treatment was ineffective. Stool culture yielded Salmonella species, and his symptoms temporarily improved with antimicrobial treatment.

In August, repeat colonoscopy showed extensive mucosal necrosis extending from the rectum to the sigmoid colon and ulcer scars in the ileocecal region (**Fig. 2A**). Epithelioid granulomas were identified in an ileocecal biopsy specimen, and stool culture was negative for Salmonella. After discharge, abdominal pain and hematochezia recurred. Colonoscopy in September showed severe rectosigmoid stenosis; stool culture again yielded Salmonella, and antimicrobial therapy produced transient improvement. He nevertheless required repeated admissions for recurrent abdominal pain, diarrhea, hematochezia, and vomiting. CT showed rectosigmoid stenosis with proximal inflammation. Double-balloon enteroscopy demonstrated longitudinal ulcer scars in the small bowel (**Fig. 2B**). By November, the rectal stenosis was impassable with the colonoscope (**Fig. 2C**) and was confirmed by contrast enema (**Fig. 2D**). He was transferred to our hospital after 5 admissions.

**Fig. 2:**
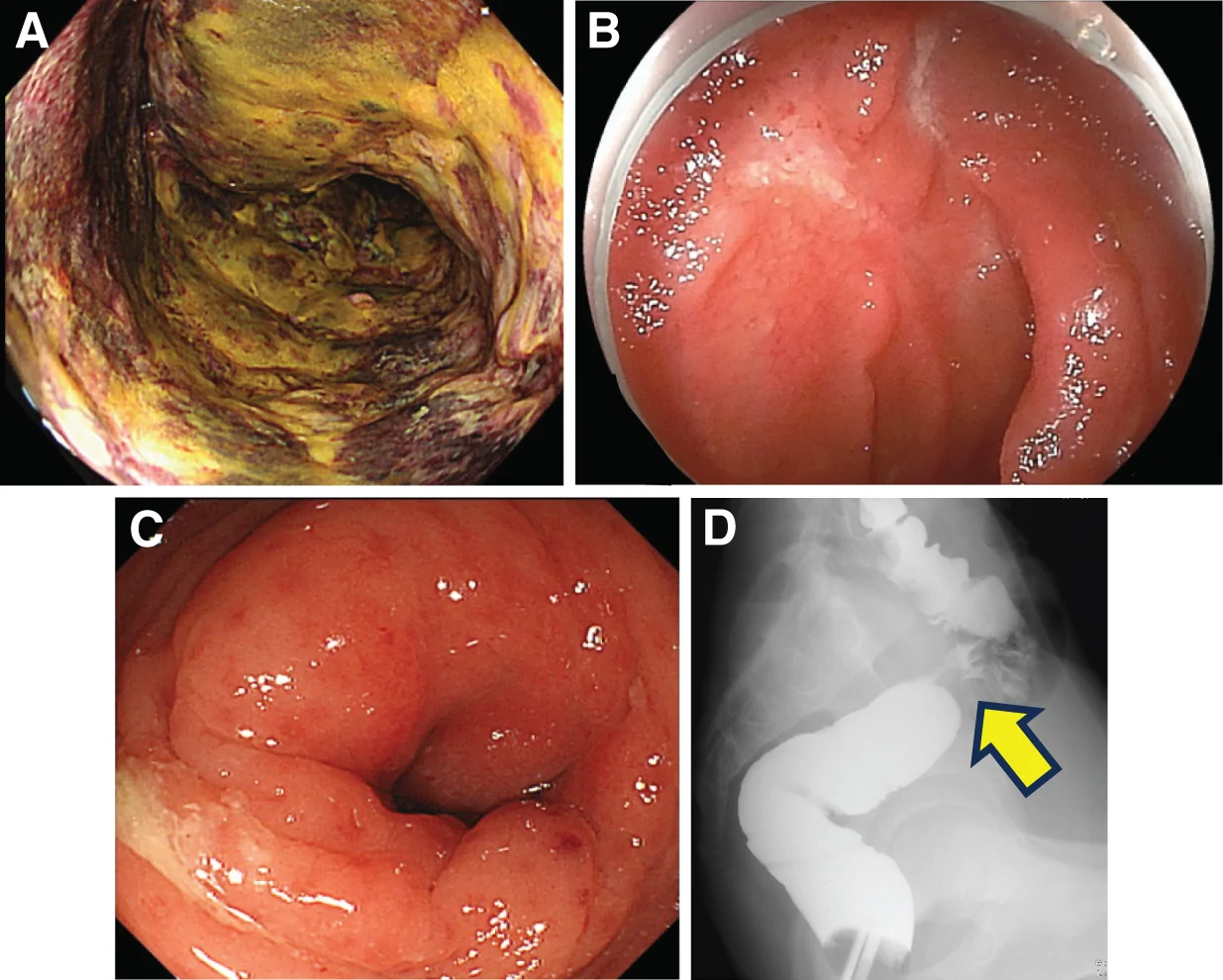
Endoscopic and radiographic findings in Twin 1. (**A**) Colonoscopy shows extensive mucosal necrosis extending from the rectum to the sigmoid colon. (**B**) Double-balloon enteroscopy shows a longitudinal ulcer scar in the small bowel. (**C**) Severe rectal stenosis prevents passage of the colonoscope. (**D**) Contrast enema shows severe rectal stenosis (yellow arrow).

On admission, his height was 170 cm and his weight was 51 kg, representing a 9-kg weight loss. His abdomen was flat and soft. Laboratory tests showed a hemoglobin level of 12.2 g/dL and a C-reactive protein level of 0.53 mg/dL; the serum albumin level was 4.6 g/dL. The combined clinical, endoscopic, and histological findings supported ileocolonic CD with stricturing behavior and perianal disease (Montreal classification: L3, B2p).

Because recurrent obstructive symptoms were attributable to fixed rectosigmoid stenosis, surgery was undertaken. The terminal ileum was adherent to the rectosigmoid colon and was carefully separated. Dense retroperitoneal adhesions necessitated conversion to an open surgery. An anterior resection with a double-stapled colorectal anastomosis was performed. Histopathological examination showed scattered epithelioid granulomas (**Fig. 3**). The postoperative course was uneventful, and he was discharged on POD 8. Vedolizumab was initiated after discharge. At more than 2 years of follow-up, he remained free of recurrent obstructive symptoms.

**Fig. 3:**
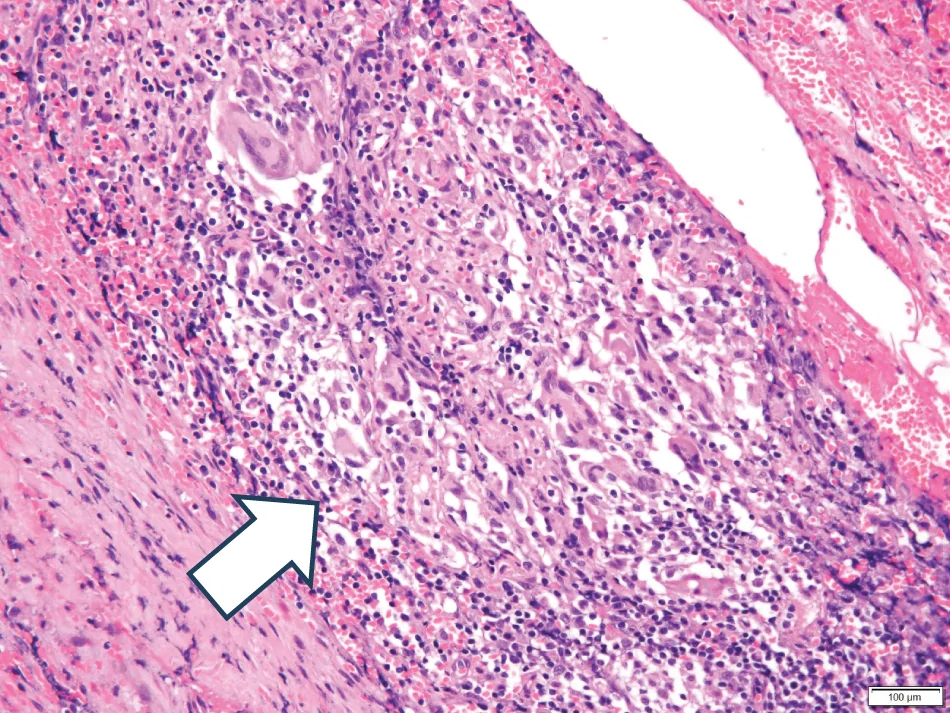
Histopathological findings in Twin 1. Scattered noncaseating epithelioid granulomas are present in the resected specimen (hematoxylin and eosin staining, original magnification ×10; white arrow).

### Twin 2

2 years later, at the age of 18 years, the younger brother developed anal pain and bleeding. A perianal abscess was incised and drained at another hospital. Because his cotwin was being treated for CD, he was referred promptly to our hospital. His height was 172 cm and his weight was 64 kg. Laboratory tests showed a white blood cell count of 10300/µL and a C-reactive protein level of 2.78 mg/dL; the serum albumin was 4.0 g/dL, and the hemoglobin level was 14.4 g/dL.

Colonoscopy showed longitudinal ulcers extending from the terminal ileum to the cecum (**Fig. 4A**) and scattered aphthous ulcers throughout the colon and rectum (**Fig. 4B**). Anorectal examination demonstrated a low intersphincteric fistula with a secondary opening at the 1-o’clock position (**Fig. 5A**). These findings supported ileocolonic CD with non-stricturing, non-penetrating behavior and perianal disease at presentation (Montreal classification: L3, B1p). Because the fistula remained painful, an examination under anesthesia was performed. Hydrogen peroxide injected through the secondary opening exited from the primary opening at the 12-o’clock position, confirming a straight tract. The tract was curetted, and loose seton drainage was established with a Penrose drain and 2 vessel loops (**Fig. 5B**). Curetted tissue contained epithelioid granulomas (**Fig. 6**). He was discharged on POD 4, and vedolizumab was initiated after discharge.

**Fig. 4:**
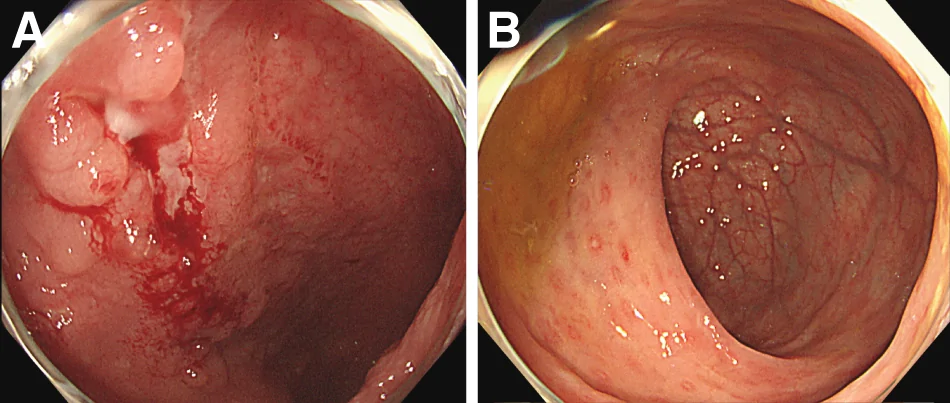
Colonoscopic findings in Twin 2. (**A**) Longitudinal ulcers are present from the terminal ileum to the cecum. (**B**) Multiple aphthous ulcers are scattered in the rectum.

**Fig. 5:**
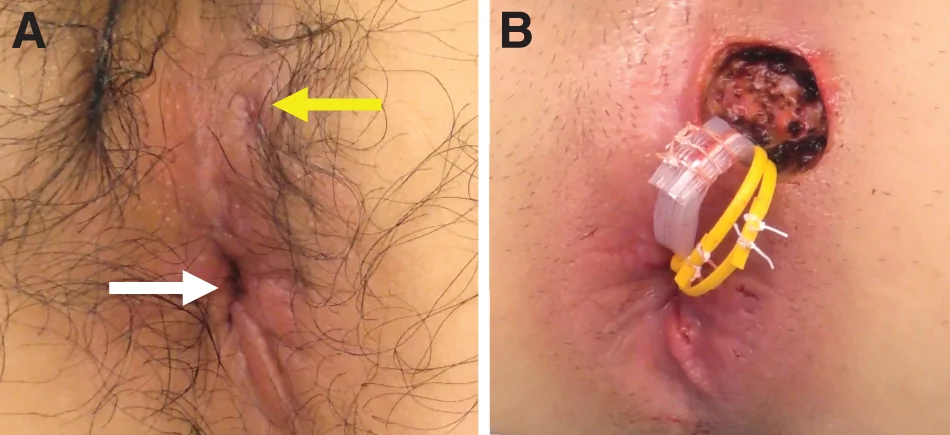
Perianal findings and operative treatment in Twin 2. (**A**) A secondary opening is present at the 1-o’clock position (white arrow, anus; yellow arrow, secondary opening). (**B**) After curettage of the fistulous tract, loose seton drainage is established with 1 Penrose drain and 2 vessel loops.

**Fig. 6:**
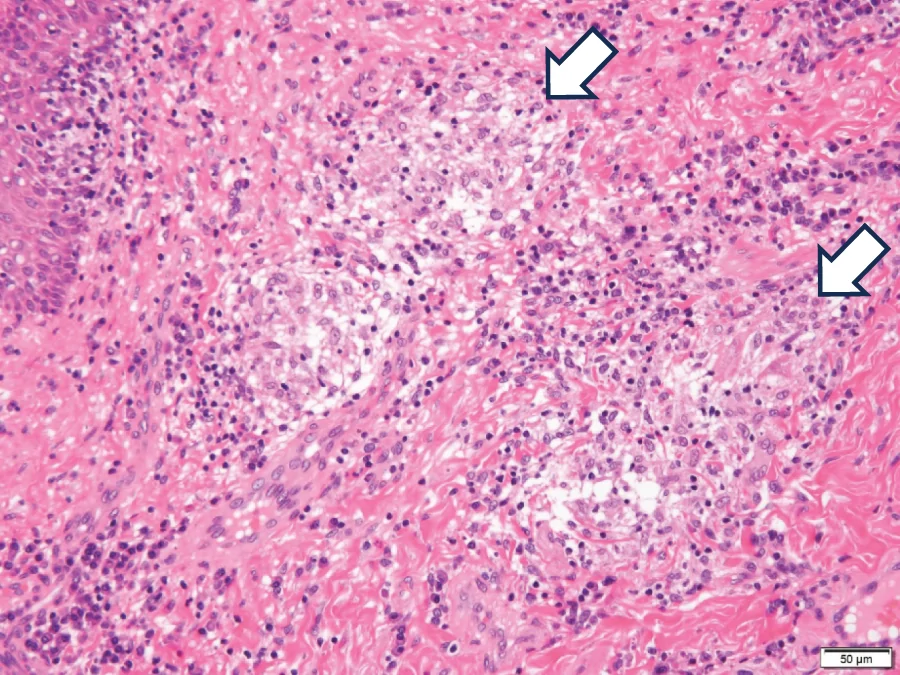
Histopathological findings in Twin 2. Noncaseating epithelioid granulomas are present in curetted fistula tissue (hematoxylin and eosin staining, original magnification ×20; white arrows).

3 months after surgery, all tubes were removed. 6 months after surgery, the perianal fistula recurred with purulent drainage; therefore, vedolizumab was switched to adalimumab. 1 month after the switch, the perianal fistula improved, and the drainage ceased. He required no additional surgery. Although this response was favorable, 7 months of postoperative observation remained insufficient to determine his long-term luminal or perianal disease course.

Selected published reports of concordant CD in twin pairs from Japan and other countries, with attention to surgical interventions, are summarized in **Table 1**.

**Table 1:** Selected concordant twin reports with surgical information

Report	Country	Onset age, years	Zygosity	Phenotype at report	Surgical treatment, twin 1/twin 2
Present report	Japan	16/18	Presumed; family report	L3, B2p/L3, B1p	Anterior resection for fixed rectosigmoid stenosis/loose seton drainage for fistula
Kimura et al. 1998^8)^	Japan	22/23	Reported monozygotic	L2, B2p/L2, B2p	No surgery/no surgery
Koh et al. 2003^9)^	Japan	19/19	Reported monozygotic	L3, B2/L1, B2B3	Ileocecal resection/ileocecal resection and right salpingectomy
Hemmi et al. 2010^10)^	Japan	12/11	Reported monozygotic	L2L4, B1/L2L4, B2p	No surgery/gastrojejunostomy
Park et al. 2010^11)^	Korea	15/15	Reported monozygotic	L3,B3/L3,B1	No surgery/no surgery
Ballester et al. 2017^12)^	Spain	20/22	Reported monozygotic	Ileocolonic and duodenal disease / same, with jejunal and perianal disease	Fistula closure, ileocolonic resection, and gastrojejunostomy/right colectomy and partial duodenectomy for coloduodenal fistulas

This is a selected, non-systematic comparison of individual concordant twin pairs. “Reported monozygotic” reflects the original report; the testing methods were not independently verified. Phenotypes and procedures for the Japanese cases were transcribed from the previously submitted table. B and L follow the Montreal classification; p indicates perianal disease.

## DISCUSSION

The principal educational feature of this report is that presumed monozygotic twin brothers both required surgical treatment for CD, but for different immediate indications. Twin 1 required bowel resection for fixed rectosigmoid stenosis and recurrent obstructive symptoms, whereas Twin 2 required seton drainage for a symptomatic perianal fistula. After all drains were removed, fistula drainage recurred and resolved after vedolizumab was changed to adalimumab, underscoring the need for continued postoperative reassessment and timely modification of medical therapy. Both brothers developed CD before the age of 20 years, with a 2-year interval between their onsets, ileocolonic and perianal involvement, and histological epithelioid granulomas. Nevertheless, treatment was determined by each patient’s contemporaneous anatomy and symptoms rather than by the cotwin’s phenotype.

An association between *NOD2* variants and susceptibility to CD also supports a genetic contribution.^5)^

Population-based twin studies support a substantial genetic contribution to CD while demonstrating incomplete penetrance. In a recent Swedish nationwide cohort of 111080 twins, proband concordance for CD was 30% in monozygotic pairs and 2% in dizygotic pairs; the corresponding values for ulcerative colitis were 15% and 3%, respectively.^6)^ The estimated heritability was 0.78 for CD and 0.57 for ulcerative colitis.^6)^ These population estimates should not be interpreted as the probability that any particular symptomatic cotwin has CD, and they do not verify the zygosity of the present brothers. Earlier cohorts reported different absolute rates owing to study design and ascertainment.^3,4)^

Recent twin-based studies have investigated mechanisms beyond inherited sequence variants. A metagenomic study reported inflammatory bowel disease (IBD)-associated microbial signatures in healthy cotwins of affected patients.^7)^ These associations do not identify the cause of disease in an individual pair. In the present patients, the histories of residence, diet, smoking, antibiotic exposure, and major infections were similar or unremarkable, but exposure data were not quantified, and no genomic, microbiome, epigenetic, or immune profiling was performed. Thus, the cause of concordance or of the different operative indications cannot be inferred.

Previously published Japanese case reports describe 3 concordant twin pairs,^8–10)^ and reports from Korea and Spain also document concordant pairs.^11,12)^ Outside Japan, concordant twin pairs have often been examined within twin cohorts rather than presented as detailed individual surgical case reports. Consequently, reports with patient-level operative information suitable for direct comparison were limited in our targeted search. These examples are compared in **Table 1**; the selection is illustrative rather than a systematic global census. In a Swedish-Danish series of 17 monozygotic pairs concordant for CD, 14 pairs developed the disease within 5 years of each other, and disease location was concordant in 11 pairs.^13)^ The Spanish pair underwent complex bowel and duodenal operations for coloduodenal fistulas in both siblings.^12)^ In contrast, our brothers required distinct operations at an early stage: resection for a fixed rectosigmoid stricture in 1 brother and drainage of a symptomatic perianal fistula in the other. The latter brother’s short follow-up precludes any claim of durable phenotypic divergence.

The surgical distinction is clinically relevant. In Twin 1, recurrent obstruction from an impassable, fixed rectosigmoid stricture despite medical treatment supported segmental resection; extensive adhesions required conversion to an open approach, and bowel continuity was restored with a colorectal anastomosis. In Twin 2, a painful low intersphincteric fistula without luminal stenosis was mapped under anesthesia, curetted, and drained with loose setons, thereby preserving the sphincter function. These decisions addressed the immediate mechanical and septic problems while postoperative biologic treatment was adjusted according to subsequent symptoms. New gastrointestinal or perianal symptoms in a cotwin should prompt targeted evaluation, as occurred after Twin 2 developed a perianal abscess. This observation does not support routine endoscopy in asymptomatic relatives.

This report has several limitations. First, zygosity was based solely on information from the patients and their family and was not objectively confirmed; the brothers should therefore be regarded as presumed monozygotic twins. Second, Twin 2 was followed for only 7 months after surgery. His classification as B1p describes his luminal phenotype at presentation and does not establish persistent divergence from Twin 1, because stricturing or penetrating complications may develop later. Third, although several relevant exposures were compared and appeared similar, the information was based on medical history and was not quantified; medication exposures other than routine antibiotic use were not systematically assessed. Consequently, the differences in surgical indications and postoperative courses cannot be attributed to nongenetic factors. Finally, the initial isolation of Salmonella in Twin 1 complicated interpretation of his severe colitis; however, recurrent inflammation and stenosis after microbiological clearance, small-bowel ulcer scars, perianal disease, and epithelioid granulomas supported a diagnosis of CD.

## CONCLUSIONS

Surgically treated ileocolonic and perianal CD developed 2 years apart in presumed monozygotic twin brothers. The known diagnosis in Twin 1 facilitated early gastrointestinal evaluation of Twin 2 when he developed a perianal abscess. The different immediate indications for anterior resection and seton drainage, followed by modification of biologic therapy after fistula recurrence in Twin 2, emphasize that management should be individualized and reassessed over time. Objective zygosity testing, more detailed exposure assessment, and longer follow-up would be required before conclusions can be drawn about genetic concordance or divergence in disease behavior.

## DECLARATIONS

### Use of artificial intelligence tools

During the preparation of this manuscript, the authors used ChatGPT (OpenAI) to assist with English-language editing and restructuring. The tool was not used to generate or analyze patient data or to modify clinical images. The authors critically reviewed and edited all output and took full responsibility for the manuscript.

### Availability of data and materials

The data supporting the conclusions of this report are included within the article. Additional patient-level data are not publicly available because of privacy considerations.
